# Bilateral Renal Artery Thrombosis in a Patient With Behçet’s Disease Managed With Infliximab Infusions

**DOI:** 10.7759/cureus.105037

**Published:** 2026-03-11

**Authors:** Osman Erhan Akcan, Sema Yilmaz

**Affiliations:** 1 Rheumatology, Selçuk University Faculty of Medicine Hospital, Konya, TUR

**Keywords:** abdominal bruit, acute kidney injury, behçet’s disease, renal artery thrombosis, secondary hypertension

## Abstract

We present this rare case of bilateral renal artery involvement due to Behçet's disease (BD) vasculitis to attract the attention of clinicians. BD is a chronic autoimmune vasculitis affecting multiple organ systems. BD causes recurrent oral and genital aphthous ulcers, uveitis, papulopustular lesions, gastrointestinal ulcers, neurologic disease, vasculitis, and arthritis. We report the case of a 56-year-old male patient with 20 years of BD who developed renal artery thrombosis, diagnosed with acute renal failure, hypertension, abdominal bruit, and thoracoabdominal computed tomography angiography images showing bilateral renal artery thrombosis. Our patient was treated with steroids, colchicine, clopidogrel, antihypertensives, and infliximab infusions with clinical and laboratory improvement. Due to active inflammation, radiologic intervention was delayed until remission. We present this case of rare bilateral renal artery vasculitis to the attention of clinicians who take care of BD patients every day.

## Introduction

Behçet disease (BD) is a chronic, relapsing, systemic, autoinflammatory disease. BD shows its highest prevalence along the ancient Silk Road, from the Mediterranean region to East Asia, likely reflecting the geographic distribution of the HLA-B51 gene [1]. BD affects the mucocutaneous, ocular, articular, vascular, neurological, and gastrointestinal systems [2]. Because no disease-specific laboratory, radiographic, or histologic markers exist, diagnosis depends entirely on clinical and phenotypic findings [3]. The worldwide prevalence of BD is estimated at 10.3 per 100,000, but Turkey has the highest known prevalence, reaching up to 420 per 100,000 [4,5]. The clinical triad of aphthous ulcers, genital ulcers, and ophthalmic involvement was originally described by the Turkish dermatologist Hulusi Behçet in 1937 [6]. More than one-third of individuals with BD develop vascular manifestations. BD can affect both venous and arterial vessels of every size. Herein, we describe a case of BD with vascular involvement presenting as renal artery thrombosis and an abdominal aortic thrombus, a combination reported in only a limited number of cases in the literature.

## Case presentation

**Figure 1:**
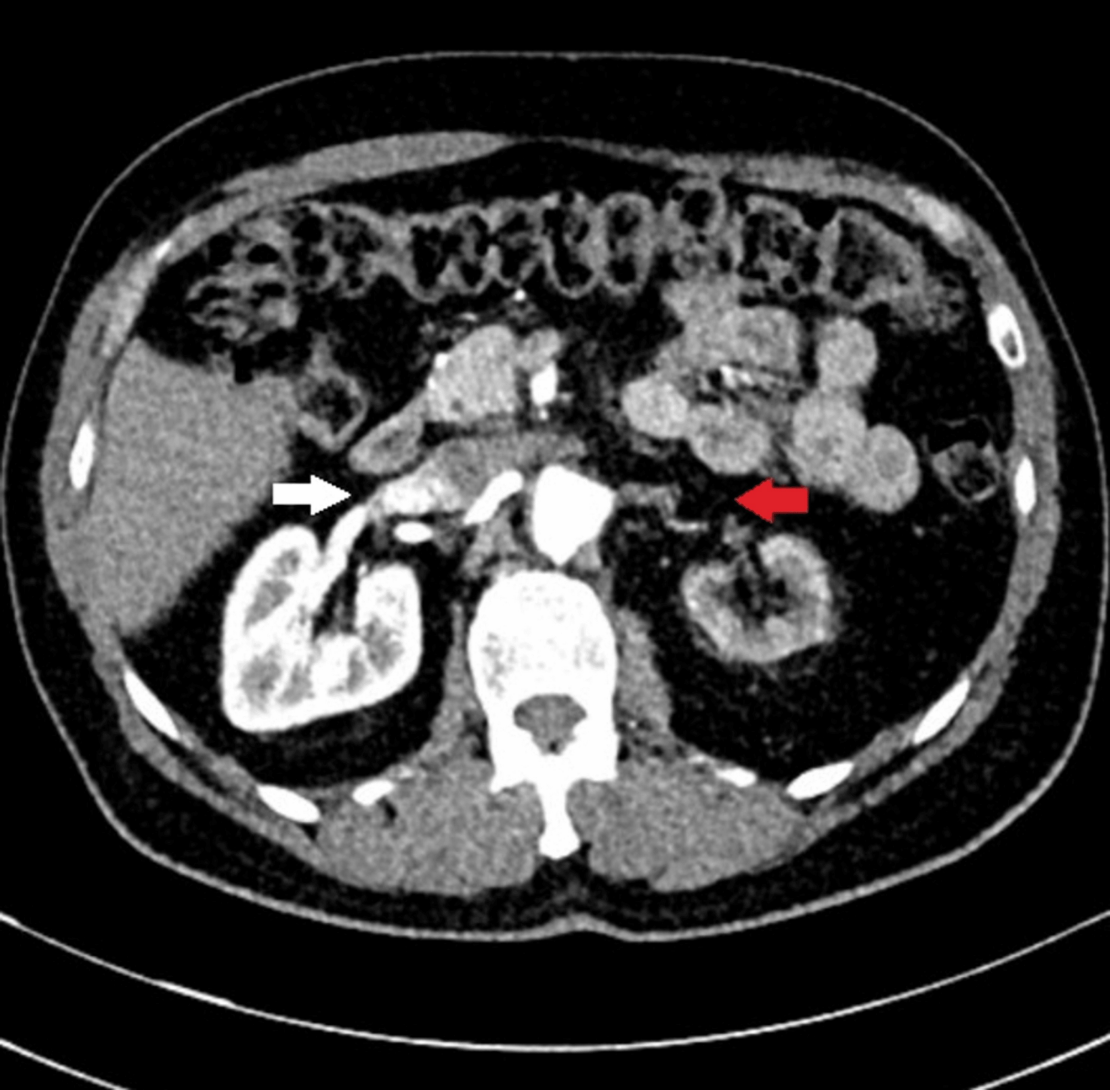
Thoracoabdominal computed tomography angiography showed 40% occlusion of the right renal artery (white arrow) and total occlusion of the left renal artery (red arrow).

**Figure 2:**
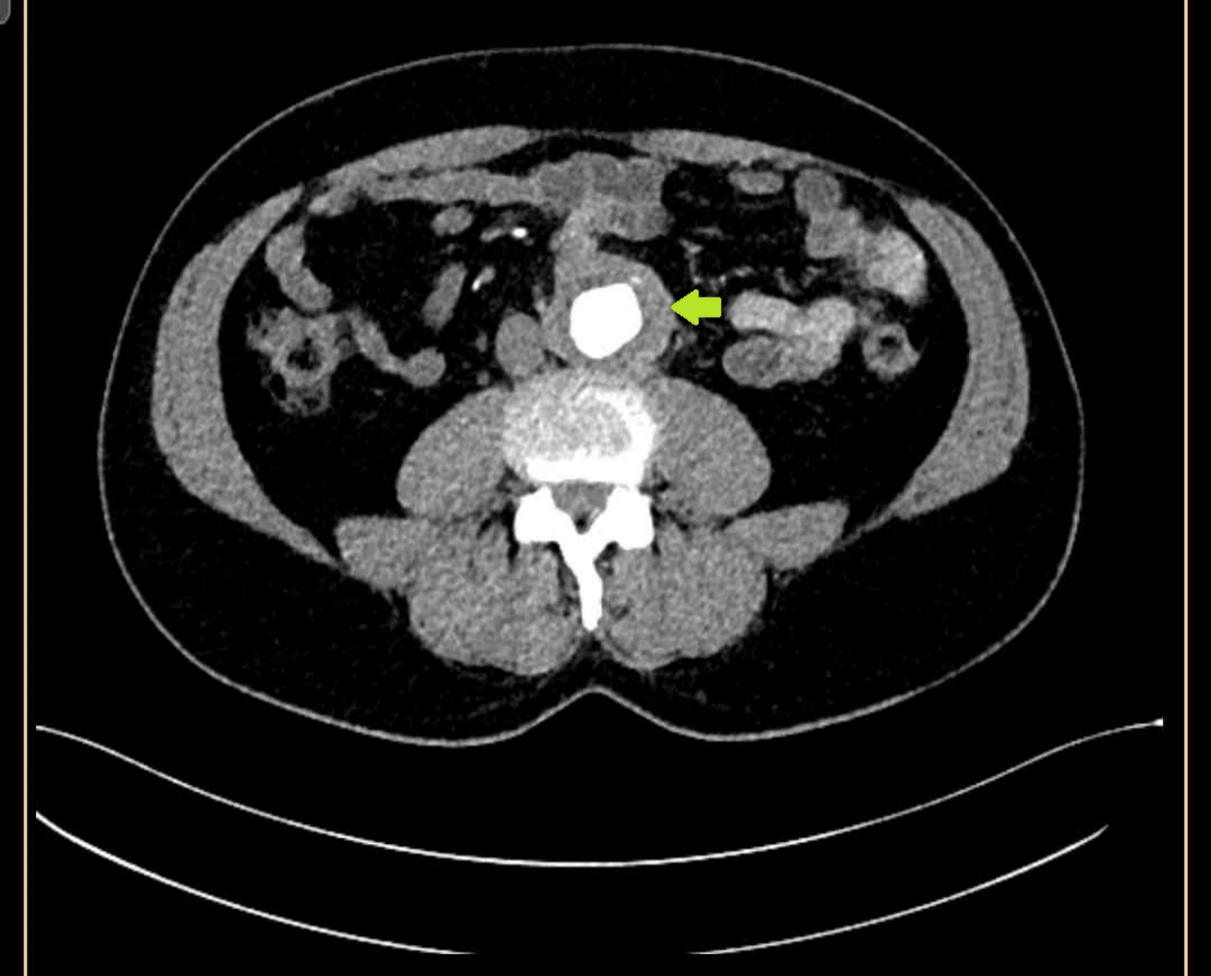
Thrombus in the aorta (yellow arrow) was noted.

A 56-year-old male patient diagnosed with BD 20 years ago, with posterior uveitis, oral and genital aphthous ulcers, and erythema nodosum, presented to the rheumatology clinic with diminished urinary outflow, decreased appetite, malaise, and abdominal discomfort. He was a nonsmoker without a history of diabetes mellitus, hypertension, hyperlipidemia, or coronary artery disease. He denied headaches, jaw and extremity claudication, morning stiffness, bloody sputum, dyspnea, night sweats, blood in urine, frequent sinusitis, rash, sicca syndromes, and paresthesias. His blood pressure was 160/100 mmHg in both arms. Peripheral pulses were palpable in all four extremities. No scalp, limb-girdle, or parotid tenderness was present. There was no sign of mononeuritis multiplex. There were aphthous ulcers of the oral mucosa, old erythema nodosum scars in the pretibial areas, and tenderness in the lower abdomen without rebound. A notable bruit in the right renal artery region was heard. His white blood cell count was 7500/ml (reference range: 4000-9750/ml), hemoglobin 13 gr/dl (reference range: 13.4-17.6 gr/dl), thrombocytes 220 k/ul (reference range: 151-387 k/ul), erythrocyte sedimentation rate (ESR): 30 mm/h (reference range: 0-18 mm/h), C-reactive protein (CRP): 10.5 mg/dl (reference range: 0-5 mg/dl), and creatinine 1.60 mg/dl (reference range: 0.7-1.2 mg/dl); his creatinine six months earlier was 0.9 mg/dl. Urinalysis showed no erythrocytes, leukocytes, or protein. His 24-hour urinary protein was 120 mg (reference range: <150 mg/24 hours). Immunological tests, including anti-nuclear antibody (ANA), extracted nuclear antigen (ENA), anti-double-stranded DNA antibody, anti-nuclear cytoplasmic antibody (ANCA), rheumatoid factor (RF), and anti-cyclic citrullinated peptide antibody (anti-CCP), were negative. Hepatitis B antigen and hepatitis C virus antibody tests were also negative (Table 1). Thoracoabdominal computed tomography angiography showed 40% to 50% narrowing of the right renal artery, allowing contrast passage, a totally occluded left renal artery, and no contrast in the left renal parenchyma (Figure 1). There was a diffuse thrombus in the wall of the abdominal aorta (Figure 2). There were no microaneurysms in the renal, hepatic, or mesenteric arteries. There was no evidence of diffuse atherosclerotic disease on imaging. His thoracic computed tomography showed no signs of a cavity or interstitial lung disease. Sputum examination and culture for tuberculosis were negative. His extensive thrombophilia and antiphospholipid antibody syndrome screening resulted in negative results. We started high-dose oral corticosteroids (1 mg/kg/day). Infliximab was initiated at 5 mg/kg intravenously (0, two, and six weeks, followed by maintenance every six weeks); azathioprine (2 mg/kg/day); colchicine (1.5 mg/day); and clopidogrel 50 mg/day after discussion with the cardiovascular surgery team. After two weeks of immunosuppressive therapy, creatinine decreased to 1.2 mg/dL, CRP decreased to 3 mg/dL, and blood pressure was controlled with antihypertensives perindopril and amlodipine. We plan to refer our patient to interventional radiology for stenting of the left renal artery after controlling inflammation with successful immunosuppressive therapy.

**Table 1:** The patient's laboratory findings

Test (unit)	Observed value	Normal range
White blood cell (/µL)	7500	4000–9750
Hemoglobin (g/dL)	13	13.4–17.6
Platelet (K/µL)	220	151–387
Erythrocyte sedimentation rate (mm/h)	30	0–18
C-reactive protein at presentation (mg/dL) / C-reactive protein after treatment	10.5 / 3	0–5
Creatinine at presentation (mg/dL) / Creatinine six weeks earlier	1.6 / 0.9	0.7–1.2
Alanine Aminotransferase (ALT) (U/L)	0.9	0.8–1.2
Antinuclear antibody (ANA)	23	Jul-40
Rheumatoid factor (RF) (IU/mL)	16	<20
Anti-cyclic citrullinated peptide (Anti-CCP) (U/mL)	7	<20
Antineutrophil cytoplasmic antibody (ANCA)	Negative	Negative
Lupus anticoagulant	Negative	Negative
Antithrombin III (%)	98	80–120
Protein C (%)	100	70–140
Protein S (%)	75	60–130
Homocysteine (µmol/L)	12	5–15
Anti-cardiolipin IgG (GPL)	4	<20
Anti-cardiolipin IgM (MPL)	16	<20
Anti-β2 glycoprotein I IgG (U/mL)	18	<20
Anti-β2 glycoprotein I IgM (U/mL)	6	<20
Paroxysmal nocturnal hemoglobinuria (PNH) clonal component test	Not detected	Not detected
Factor II mutation	Not detected	Not detected
Factor V Leiden mutation	Not detected	Not detected
JAK2/CALR/MPL mutation	Not detected	Not detected

## Discussion

BD is a chronic, multisystem inflammatory disease that can cause vasculitis and organ damage. Although the exact pathogenesis of BD is unknown, it is largely driven by neutrophil hyperactivation. This hyperactivity is associated with HLA-B51 and is mediated by the activation of Th1 cells that secrete IFN-γ and IL-12, and of Th17 cells that secrete IL-17 and other cytokines [7]. The IL-23/IL-17 axis also plays an important role in maintaining chronic inflammation [8].

Nearly 40% of BD patients experience vascular disease, and they are usually males with active disease [9]. Our patient had oral aphthous ulcers for one month. Seventy-five percent of vascular events occur within five years of disease onset [10]. Our patient was diagnosed 20 years ago. Both arteries and veins can be affected. Venous involvement is observed in 15% to 40% of patients; arterial involvement is observed in 3% to 5% of patients [11]. Our patient did not have venous involvement but had oral and genital aphthous ulcers, erythema nodosum, and posterior uveitis. HLA-B51 positivity is associated with more severe disease, especially vascular involvement [12]. Our patient was identified as HLA-B51 positive. Ocular involvement in BD is associated with more frequent vascular involvement [13]. Our patient had prior posterior uveitis. Arterial involvement can cause occlusion, stenosis, and aneurysms; our patient had both renal artery occlusion and an abdominal aortic thrombus. Extrapulmonary arterial involvement usually occurs five to 10 years after disease onset, as in our patient [14]. The prognosis for patients with arterial involvement is poor, and pulmonary artery aneurysm rupture significantly increases the risk of death [15]. BD rarely affects the kidneys, and renal artery thrombosis is especially rare. Only a limited number of cases have been reported in the literature [2]. Invasive procedures, such as conventional angiography, can induce thrombosis or the development of pseudoaneurysms; therefore, noninvasive imaging methods, such as color Doppler ultrasonography, computed tomography angiography, and magnetic resonance angiography, are typically recommended for diagnosis [16].

The diagnostic process begins with a thorough anamnesis and continues with a thorough physical examination. We examined our patient's subclavian, carotid, and renal arteries for bruits. We heard a loud bruit over the right renal artery. The presence of a high-grade abdominal bruit raised suspicion for significant renovascular stenosis, so we ordered a CT scan to evaluate for renal artery thrombosis. Our patient’s computed tomography angiogram was compatible with narrowing of the right renal artery and total occlusion of the left renal artery.

Our patient presented with high blood pressure. Although performed in children, a review by Wang et al. found that high blood pressure is a prominent feature of renal artery lesions in BD [17].

Takayasu arteritis can affect the renal arteries. Akkuzu et al. described a BD patient with subclavian and left renal artery involvement resembling Takayasu arteritis [18]. Our patient has no blood pressure difference between arms; his pulses were palpable in all four extremities, and there was no evidence of vasculitis in the subclavian arteries.

Another vasculitic disease that can affect the abdominal aorta and renal arteries is temporal arteritis (TA). We know that TA typically affects individuals over 50 years of age; however, the absence of scalp tenderness, jaw and extremity claudication, morning stiffness, headaches, and a sedimentation rate below 50 mm/h makes TA unlikely.

Granulomatosis with polyangiitis (GPA) and microscopic polyarteritis nodosa (mPAN) can affect the abdominal aorta and renal arteries. The absence of hematuria, hemoptysis, significant proteinuria, active urinary sediment, weight loss, frequent sinusitis, paresthesias, interstitial lung disease, lung nodules and cavities, and a negative ANCA test makes GPA and mPAN unlikely.

PAN can affect the same vessels; however, the absence of microaneurysms in the renal, mesenteric, and hepatic arteries, negative hepatitis B serology, absence of mononeuritis multiplex, livedo reticularis, and testicular involvement make PAN unlikely.

Tuberculosis infection can cause aortitis; however, the absence of hemoptysis, weight loss, night sweats, and an apical lung cavity, along with negative sputum microscopy and culture for tuberculosis, made the diagnosis unlikely.

Thrombophilia and antiphospholipid syndrome (APS) can cause renal artery thrombosis. Our patient's antithrombin III, factor II, V Leiden mutations, C and S proteins, homocysteine, anti-cardiolipin IgM and IgG, anti-beta-2-glycoprotein I IgG and IgM, lupus anticoagulant, paroxysmal nocturnal hemoglobinuria clonal component test, and JAK2-CALR-MPL mutation were all negative.

Cyclophosphamide is the standard remission induction therapy for severe BD [19]. The toxicity of cyclophosphamide is well known. It can cause secondary malignancies, infertility, and pancytopenia, but a recent randomized head-to-head trial by Saadoun et al. showed that infliximab is superior to cyclophosphamide for induction of remission in BD with vascular or neurological involvement; 81% of patients receiving infliximab achieved a complete response, compared with 56% of patients receiving cyclophosphamide [20]. Another study by Hatemi et al., involving 127 vascular BD patients who had received adalimumab, azathioprine, and cyclophosphamide previously, reported a 73% remission rate with infliximab at six months and a 63% rate at 12 months [21]. Based on these studies, we treated our patient with oral steroids, infliximab, colchicine, azathioprine, and clopidogrel. Conventional angiography was not performed due to the potential risk of procedure-related vascular complications in BD.

## Conclusions

Renal artery thrombosis in BD is rare. Conditions other than BD that cause renal artery thrombosis must be excluded. After exclusion and imaging studies demonstrate renal artery thrombosis in a patient with BD, the condition can be treated according to recent literature. The general trend is toward the use of cyclophosphamide; however, recent studies indicate that infliximab is both more effective and associated with fewer adverse effects. This case highlights the importance of meticulous vascular examination in BD. It emphasizes that renal artery thrombosis, although rare, should be considered in BD patients presenting with hypertension and an abdominal bruit. Early recognition and prompt immunosuppressive therapy may prevent catastrophic vascular complications.
